# Challenges in Bridge Health Monitoring: A Review

**DOI:** 10.3390/s21134336

**Published:** 2021-06-24

**Authors:** Piervincenzo Rizzo, Alireza Enshaeian

**Affiliations:** Laboratory for Nondestructive Evaluation and Structural Health Monitoring Studies, Department of Civil and Environmental Engineering, University of Pittsburgh, 3700 O’Hara Street, 729 Benedum Hall, Pittsburgh, PA 15261, USA; ale69@pitt.edu

**Keywords:** structural health monitoring, sensors, nondestructive evaluation, bridges, state-of-the-art review

## Abstract

Bridge health monitoring is increasingly relevant for the maintenance of existing structures or new structures with innovative concepts that require validation of design predictions. In the United States there are more than 600,000 highway bridges. Nearly half of them (46.4%) are rated as fair while about 1 out of 13 (7.6%) is rated in poor condition. As such, the United States is one of those countries in which bridge health monitoring systems are installed in order to complement conventional periodic nondestructive inspections. This paper reviews the challenges associated with bridge health monitoring related to the detection of specific bridge characteristics that may be indicators of anomalous behavior. The methods used to detect loss of stiffness, time-dependent and temperature-dependent deformations, fatigue, corrosion, and scour are discussed. Owing to the extent of the existing scientific literature, this review focuses on systems installed in U.S. bridges over the last 20 years. These are all major factors that contribute to long-term degradation of bridges. Issues related to wireless sensor drifts are discussed as well. The scope of the paper is to help newcomers, practitioners, and researchers at navigating the many methodologies that have been proposed and developed in order to identify damage using data collected from sensors installed in real structures.

## 1. Introduction

The growth of the world’s population increases the tonnage of commodities and the volume of private and public vehicles that cross bridges worldwide. Some of these bridges may incorporate innovative materials whose degradation processes are not well known. In the U.S., the evaluation of bridges starts with a periodic inspection, typically a visual inspection, conducted in accordance with the National Bridge Inspection Standards. When a given bridge shows problematic areas, it may be inspected more frequently at the discretion of the owner using advanced tools such as ultrasounds or ferromagnetic methods, just to mention a few.

As traditional nondestructive evaluation (NDE) maintenance can do little when flaws start or become critical between two inspections, there is a growing interest in cost-effective structural health monitoring (SHM) strategies. SHM shifts the maintenance paradigm from “time-based” to “permanent-based” where a network of sensors monitor the structure of interest 24/7 in order to flag, locate, and quantify damage as it happens [1,2,3,4,5,6]. Besides the scope of detecting damage at the earliest possible stage, reliable SHM systems may monitor certain bridge parameters to assess a bridge’s performance under various service loads, to verify or update the rules used in its design stage, and to prioritize maintenance and rehabilitation. In 2011, Xu and Xia [7] listed nine major bridges (Table 1) in the U.S. equipped with health monitoring systems. Nearly ten years later, the authors of this paper identified at least 60 bridges in the U.S. with active or discontinued SHM programs in addition to those listed in Table 1 (see Table 2 of ref. [8]).

In any bridge health monitoring, sensors directly or indirectly measure external loading (wind, seismic, and traffic), structural responses (strain, displacement, and acceleration), environmental parameters (temperature, humidity, and rain), and environmental effects (corrosion). The sensors are connected to dedicated hardware/software for storage and, ideally, for real-time assessment. In this article, the discussion of the issues related to the detection and processing of physical parameters for the health monitoring of bridges is discussed. This paper complements the work published by the authors [8] in which a thorough review of the scientific literature of the structural health monitoring systems installed in U.S. bridges over the last 20 years was presented. That review aimed to offer interested readers a holistic perspective of recent and current state-of-the-art bridge health monitoring systems and to extract a “general paradigm” that is common to many real structures. In this paper, the issues related to the physical parameters considered as damage precursors or damage indicators and the challenges associated with the health monitoring of bridges including the drift of wireless sensing are presented. Some of the most important factors that degrade a bridge are discussed from the perspective of their detectability with the sensors. Most of the discussion is about U.S. bridges. However, some case studies from Europe and Asia are presented. This review is organized by the type of information to be gathered from the health monitoring systems. Data inference methods used to detect loss of stiffness, time-dependent (creep and shrinkage) and temperature-dependent deformations, fatigue, corrosion, scour, and accidental impacts are included as they are all considered major factors that contribute to long-term degradation of bridges.

## 2. Parameters Affecting Bridge Condition

Modares and Waksmanski [9] sorted SHM sensing systems by parameters and provided details of sensor types, accuracy, range, and operating temperature. The considered parameters were (in alphabetical order): corrosion, cracking, displacement, fatigue, force, settlement, strain, temperature, tilt, vibration, water level, and wind. In addition, they classified the types of sensors as either contact or noncontact. With progress in technology, new sensing capabilities are developed and two excellent reviews on the subject were published by Sharyatpanahi [10] and Moreno-Gomez et al. [11], while a review focusing on sensors for concrete monitoring was presented by Taheri [12].

Data inference is a critical part of any bridge health monitoring because diagnostics and prognostics will be eventually made based on the processing of the data streamed from different parameters (sensors). For bridge condition evaluation and prediction, both short and long-term factors should be considered. The analyses conducted by the authors [8] made evident that most SHM implementations do not rely on a single nondestructive evaluation method, e.g., strain measurements, because an SHM protocol based on a single parameter is not able to monitor all factors that are critical to a bridge. As such, integrated systems that contain different sensor types are warranted.

### 2.1. Stiffness Loss

The loss of stiffness in any given structural component is considered by many a reliable indicator of damage. As such, several methods were proposed for the detection and localization of stiffness losses. Some of the localization methods rely on the detection of irregularities in the deflected shape of the structure [13,14,15,16]. These methods are based on the determination of the modal characteristics of the structure and in particular on the accurate determination of the deflected shape. This can be achieved by using a high spatial resolution of sensors, high quality measurements, and reliable signal processing. One of the major advantages of vibration-based damage identification methods is the possibility of detecting damage at a global level using sensors not necessarily deployed close to the location of damage, which is typically unknown. The estimation of stiffness loss can be made by using response-only approaches, which are based on the use of sensor data only, and/or by using physical-based models, such as finite element models.

Limongelli [17] reviewed the vibration-based damage localization algorithms based on the detection of (changes of) irregularities in deflected structural shape. The review focused on methods that perform localization through the detection of irregularities in the deflected shape of the structure. Most of these methods exploit the relationship between a local loss of stiffness and the corresponding local variation of curvature. The latter, which becomes the damage-sensitive feature, requires double differentiation of the displacement data obtained from a dense and distributed network of sensors. Unfortunately, the use of dense arrays of sensors drives up the cost of the SHM. Another drawback is related to the estimation of curvature from noisy recorded responses. To mitigate these drawbacks, some authors proposed methods to identify variations of curvature without explicitly computing curvatures or through numerical validation using finite element approaches, due to the limited availability of experimental data directly associated with damaged structures. Recently, data recorded on benchmark structures have become available giving the opportunity to verify the capability of these methods for damage localization in real-world conditions.

The interested readers are referred to [17] to learn more about individual methodologies based on modal and operational shapes, shape variation due to a loss of stiffness, methods based on curvature, and methods based on the indirect detection of curvature changes.

### 2.2. Time and Temperature-Dependent Factors

Time-dependent and temperature-dependent deformations have been a concern for decades because creep and shrinkage affect concrete structures over time, whereas thermal strain and thermal stress may mask damage-related effects and live load disturbances.

**Figure 1:**
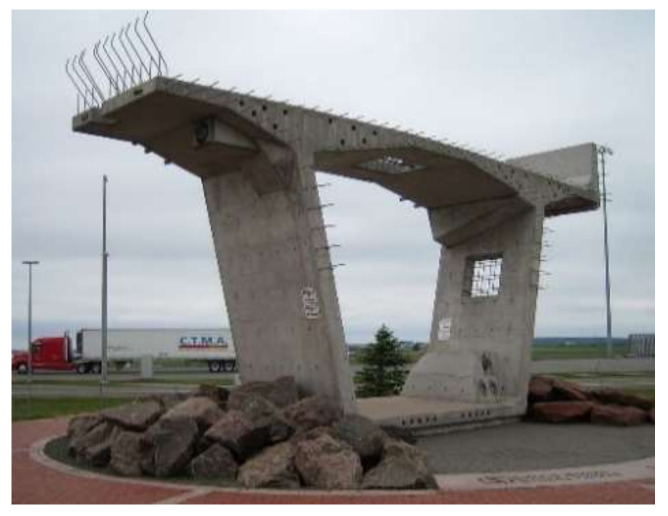
Display of a typical segment of the Confederation Bridge. (https://en.wikipedia.org/wiki/Confederation_Bridge#/media/File:188_-_Piece_of_the_Confedration_Bridge.JPG, accessed on 23 June 2021).

A number of models were proposed to predict temperature effects and to predict time-dependent factors such as sustained live load. Ghali et al. [18] investigated both short-term and long-term behavior and performance of the Confederation Bridge in Canada. This bridge is a 12.9 km structure made of box girders, such as the one on display in Figure 1. The study analyzed the time-dependent properties of concrete and compared field measured deflections to predicted deflections. Creep was estimated using CEB–FIP MC90 and ACI (American Concrete Institute) 209 codes. Ten-cylinder creep tests were conducted by using the least square fitting of the measured creep coefficients; a best-fit predictive creep equation was developed. For shrinkage, six (6) cylinders were taken to measure the free shrinkage. Measured shrinkage strain was used to obtain the predictive shrinkage equations. With other material parameters, creep and shrinkage equations were employed to analyze the deflections. After the analyses of long-term deflection, all the analyses were conducted again to induce the variation of temperature during the same time intervals. The result showed that with consideration of temperature effects, the predicted results became closer to the measured ones.

Robertson [19] presented the results of nine years of vertical deflection monitoring data of the North Halawa Valley Viaduct. The author found a disagreement between the theoretical design predictions and measured vertical deflections, and proposed an improved creep and shrinkage model, as it was believed that the sensors’ data were reliable. In support of the improved creep and shrinkage models, numerous laboratory tests were conducted and four existing predictive models were considered: the ACI 209 model, the CEB-90 model, the short form of Bazant B3, and the Gardner model. A conclusion of the study was that the short form of the Bazant B3 model predicts long-term creep best, whereas the Gardner model predicts the long-term shrinkage best. These two models were combined and applied to the North Halawa Valley Viaduct. The outcome was the agreement between the model predictions and the sensors’ field data.

Bazant et al. [20] investigated excessive deflections of Koror-Babeldaob Bridge in Palau. They compared many models including the ACI Committee 209 (American Concrete Institute (ACI) 1992), B3 model [21,22,23], CEB-FIP Model Code (CEB-FIP 1990), GL2000, and the JSCE Japanese Code (JSCE 1991). The research revealed that most of the models that were current at that time underestimated the deflections. Model B3 seemed to be the best because the multiple calibrated independent material parameters could fit the measured deflection well by linear regression scaling.

Sousa et al. [24] conducted a long-term assessment of the Leziria Bridge in Portugal using the European Code 2 (EC2) (European Committee 2004). With assumed design material parameters and computed creep/shrinkage functions, finite-element models of the Bridge were created. The results showed that time-dependent behaviors based on the fitted models could satisfy the measured trends in first five years.

**Figure 2:**
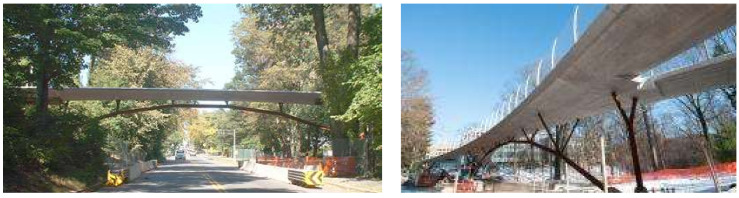
Photos of the Streicker Bridge at Princeton University, United States (Left: [26]; Right: [27]).

Glisic [25] developed a model to predict creep and shrinkage in the Streicker Bridge (Figure 2). The model is periodically updated using field measurements taken from fiber optics sensors embedded in the bridge at the time of construction.

Temperature gradient may significantly affect the static and dynamic characteristics of bridges overall or bridge components. Catbas et al. [28] developed a reliability model for the Commodore Barry Bridge in Chester, Pennsylvania considering dead load, wind pressure, traffic loads, temperature effects, and their combinations. The data came from vibrating-wire accelerometers, strain sensors, weigh-in motion devices, and tiltmeters installed in the late 1990s [29]. The health monitoring strategy proposed by Catbas et al. [28] aimed to minimize the uncertainties related to phenomena which are difficult to model. One of the findings was that temperature-induced stresses on critical elements are not very easy to conceptualize and subsequently model. It was observed that the truss elements experience bending strains due to temperature. The peak-to-peak strain differential was observed to be around 400 με, which was about ten-fold higher than the maximum strains induced by traffic. Another outcome was that thermally-induced strains in this bridge cannot be neglected in any reliability estimation model.

Jin et al. [30,31] combined a vibration-based damage detection method and extended Kalman filter-based artificial neural network (EKFNN) to eliminate the temperature effects and detect damage in a single-span 26-m long bridge in Meriden, Connecticut. The structure has multiple plate stringers supported by eight girders. Jin and co-authors [30,31] used vibration acceleration and temperature data obtained from the bridge to identify and analyze the correlations between natural frequencies and temperature in order to select proper input variables for the neural network model. One-year-long monitoring data were used to train the network. Structural damage scenarios were simulated in a finite element model under SAP2000. The damage indicator was the change in the ratios of natural frequency. In the testing phase, the damage simulation data of natural frequency time series were presented to the trained model, and the occurrence of damage was successfully detected by the control limits provided by the damage detection model. The results of the neural network indicate that the EFKNN has better capabilities than the benchmark multiple linear regression approach.

Temperature compensation methods were proposed in [32,33] and applied to the Tacony-Palmyra Bridge. A temperature-based baseline was developed by minimizing the local effects of temperature gradients and local bending using field data collected for three years. The bridge, which is a combination steel tied-arch and double-leaf bascule bridge, and includes a 168-m steel tied-arch span and a 79-m bascule span, was equipped with a wide variety of sensors and hardware. Yarnold et al. [32,33] used this bridge as a testbed of a temperature-based structural identification technique in which temperature is the forcing function, local strains are another input, and the global displacements are the outputs. The objectives of the evaluation included: (1) FEM calibration, (2) long-term performance, and (3) development of automated alert criteria. Yarnold and Moon [34] used the relationship between temperature changes and the consequent strains and displacements to create a graphical baseline of the bridge for SHM purposes. They found that the nonlinear relationship between temperature, local mechanical strains, and global displacements results in a near-flat surface when plotted in 3D space.

Similarly, Hedegaard et al. [35,36,37] applied a method to extract the time-dependent behaviors from the field monitoring data in a varying-temperature environment for the St. Anthony Falls Bridge.

Besides the above cases, which are relative to U.S. bridges, a work by Peeters et al. [38] and relative to a bridge in Leuven (Belgium) is noteworthy. They proposed a methodology for vibration-based damage detection under varying temperature. They removed the effect of the temperature from the identified vibration frequencies using linear regression analysis, specifically the ARX model. Norouzi et al. [39] created a statistical model for the U.S. Grant Bridge connecting Portsmouth, Ohio and Fullerton, Kentucky based on temperature variations during long-term SHM of the bridge. They exploited the existing correction between the strain reading from the strain gages and the temperature variation to establish a model that could describe the normal behavior of the bridge. Temperature effects were discriminated using classical curve fitting methods. An analogous method was applied in [40] to investigate the abnormalities in the behavior of the Ironton–Russell Bridge between Ironton, Ohio and Russell, Kentucky. They used the autoregressive integrated moving-average model (ARIMAX) to represent the strain gage data as a function of the temperature. This model was able to detect a drastic change in the strain gage readings in mid-2014.

The ambient temperature not only affects the strain gage reading but is also a significant parameter that can alter dynamic properties of the bridges. In this regard, Zolghadri et al. [41] investigated the influence of thermal variations on the natural frequencies of three different bridges in Utah and California. In that work, the linear autoregressive model with exogenous terms (ARX) was applied to establish a relationship between the temperature and the measured resonant frequencies. Moreover, they determined the number of appropriate inputs to their ARX model based on the principal component analysis (PCA) of the data.

Recent investigations are also exploiting statistical methods such as the PCA to recognize the individual influence of different types of loads such as thermal, wind, and traffic on the structural response of the bridges [42,43,44]. Huang et al. [44] applied a temperature-strain correlation model to eliminate the thermal effects on the strain. Moreover, they used the PCA technique to distinguish permanent trends in the data from the traffic and wind loads. Zolghadri et al. [41] applied the PCA to estimate the optimum number of input variables for their ARX strain-temperature model.

Omenzetter and Brownjohn [45] studied the Singapore–Malaysia Second Link. Strain data were modelled using a univariate model that described the signal recorded by a single strain sensor and using a multivariate model. The latter enabled the analysis of signals from multiple channels and took into account the correlation among the signals. The method was applied to strains recorded during the construction of the bridge when the structure underwent significant changes such as those related to the tensioning of tendons. After inauguration, the same two models were used to analyze real strains from daily service. During the analysis, some changes were observed. As the proposed approach did not use temperature data, the authors argued that the changes were caused by abnormal temperature variations and not structural changes.

### 2.3. Fatigue Evaluation

Understanding and predicting the fatigue behavior of bridges is important especially in lieu of aging infrastructures. Any initial fatigue crack may propagate due to an increase in traffic tonnage, harsh environment, design errors, and age. Several researchers have proposed different mathematical tools to assess and evaluate bridge fatigue reliability. In this section, a few studies are briefly summarized.

Li et al. [46] assessed damage and predicted the lifespan of bridge-deck sections of existing bridges using SHM strain-history data and a fatigue damage model based on the continuum damage mechanics. Bridge-deck structures were modeled with elastic members and welded connections with possible accumulative damage. To gage the reliability of the proposed approach, a modified Palmgren–Miner rule was developed for the same fatigue problem.

Zhou [47] proposed a procedure for fatigue life evaluation of existing bridges based on field-measured strain data. An AASHTO fatigue evaluation method was reviewed and compared with the proposed procedure. Three bridges were used as case studies (the Cleveland Central Viaduct over the Cuyahoga River in Cleveland (OH), the I-95 bridge over James River located in Richmond (VA), and the U.S. 13 bridge over Pocomoke River in Pocomoke City, Maryland). Fatigue life was evaluated based on the field-measured stress range histograms under traffic load. With analyzed results from the three bridges, conclusions were drawn to make an evaluation procedure for fatigue life of bridges.

Liu et al. [48] tested an approach to assessing bridge performance through a series-parallel system modeled on the I-39 Wisconsin River Bridge. Strain data from an SHM were used along with data from actual traffic. The sensitivity of the model was evaluated by using the actual SHM data collected in 2004. They concluded that the system reliabilities of the bridge can be predicted by using the component performance function and sensitivity studies developed under this study.

Kwon and Frangopol [49] applied concepts related to fatigue reliability to the Neville Island Bridge and the Birmingham Bridge, both in the city of Pittsburgh, Pennsylvania. Probability density functions were used to estimate equivalent stress ranges based on field data. In addition, the AASHTO S-N curve was used to provide relevant information about structural details. Lognormal, Weibull, and Gamma distributions were considered. The rain-flow counting method was used to obtain the stress-range bin histogram from the monitoring data. There were seven steps in total to conduct the assessment of the two bridges.

The Tsing Ma Bridge in Hong Kong, the world’s 14th longest span suspension bridge and the 2nd longest at time of completion, was the subject of several studies related to the estimation of fatigue life. The bridge has two decks and carries both road and rail traffic. Chen et al. [50] proposed a fatigue analysis for this bridge using data from the instrumented SHM systems. The framework was then generalized to include any long-span suspension bridges. Dynamic stress analysis was conducted and maximum stress range was selected as the index to identify the fatigue-critical locations of bridge components. The database of wind-induced, railway-induced, and highway-induced dynamic stress response was established based on the site measurements. A rain-flow counting method was used in the fatigue analysis. For the same bridge, Ye et al. [51] developed a fatigue life assessment method based on strain data and then stress-time histories obtained by converting strain data. The rain-flow counting algorithm and statistical analysis were used to identify the standard stress spectrum. Fatigue life was calculated by using the S-N curve method and Miner’s rule. Ni et al. [52] proposed a fatigue assessment method to integrate the hot spot stress range which was based on the Miner’s damage cumulative rule with continuous probabilistic formulation. In this study, field-measured data and stress concentration factors were considered as random variables to develop a probabilistic model for fatigue life evaluation. The stress range from monitoring data was created by using the finite mixture distribution and a hybrid parameter estimation method.

Guo et al. [53] proposed an approach to evaluate the time-dependent fatigue reliability of steel bridges with traffic load model and probabilistic finite element analysis. An equivalent stress range was obtained by integrating collected weigh-in-motion data and finite element analyzing under uncertainties. By regression analysis, the most appropriate probabilistic distribution of equivalent stress range was determined. This fatigue reliability assessment of steel bridges subjected to fatigue cracking was applied to the Throgs Neck Bridge, a suspension bridge built in 1961 in New York City.

**Figure 3:**
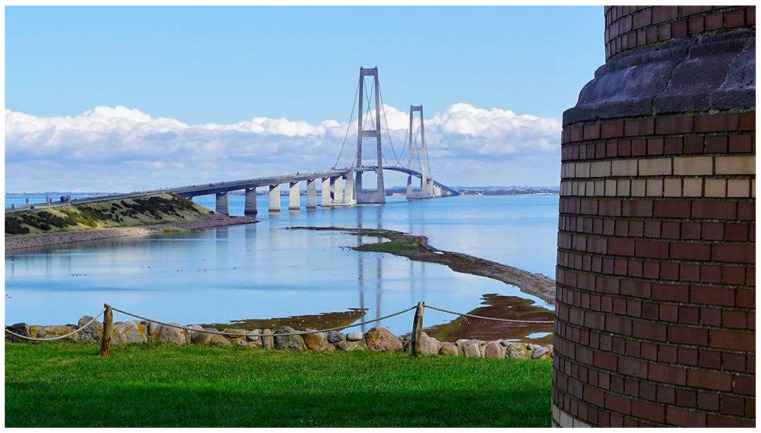
The Great Belt Bridge in Denmark [55].

Farreras-Alcover et al. [54] proposed a fatigue reliability evaluation method for welded joints of orthotropic bridge steel decks. The method used real data to characterize pavement temperatures and heavy traffic counts by means, respectively, of autoregressive models for the case of pavement temperatures and autoregressive models combined with regression models for traffic intensities. The method was illustrated by analyzing the data of field-monitoring measurement from the Great Belt Bridge in Denmark (Figure 3). The result revealed that the time of reaching a nominal target reliability was reduced by 27% with consideration of pavement temperature and heavy traffic cases.

### 2.4. Corrosion Evaluation

Corrosion in metallic parts such as cables, reinforcements, connections, or girders may degrade bridge performance. Monitoring corrosion is therefore necessary to identify critical degradation that needs maintenance. Over the last 20 years, some researchers have investigated this topic. Morris et al. [56] investigated the effects of local variables on rebar corrosion process and proposed a criterion for rebar corrosion evaluation based on measurements of concrete electrical resistivity. Two exposure conditions, namely seashore environment and partial immersion in a saline solution, were selected. Two water-to-cement ratios and various initial chloride ion additions were selected for the experiment. The results showed that the electrical resistivity can be used to evaluate the potential of steel corrosion. Additionally, concrete mix design, environmental exposure conditions, and initial chloride concentration have an effect on rebar corrosion process. No specific bridges were monitored or tested as part of this study.

Deeble Sloane et al. [57] proposed a strategy to monitor the eventual corrosion of the high-strength steel wires of suspension bridges. The strategy is based on a sensor network that assesses indirectly the environmental conditions and deterioration of the main cables. The strategy was tested on a full-scale mock-up cable recording temperature, relative humidity (RH), and corrosion rate levels. The tested sensor network was able to provide suitable clues about the interior environment of the cable. Although the observed trend was not consistent throughout the cross section of the mock-up cable, the RH values were strong indicators of corrosion rate levels. The same group later applied the same strategy on the Manhattan Bridge [58,59]. The field data showed that corrosion levels increased with the relative humidity level increasing, and relative humidity did not vary with cable depth. It is noted here that detection of corrosion in bridge structures is quite a significant issue and the fact that the scientific literature is not as rich as for other issues shall not mislead the reader. Problems with corrosion losses and diagnostics on pre-stressed rebar or post-tensioned tendons, for example, exist and are typically addressed by using conventional or advanced nondestructive evaluation methods.

### 2.5. Scour

Scour is the erosion or removal of stream bed or bank material around bridge foundations due to flowing water. Excessive scour can cause bridges to become unstable and therefore unsafe for traffic [60]. Scour monitoring devices can be clustered in fixed and portable groups. Fixed devices include sonars, magnetic sliding collars, float-out devices, sounding rods, tilt sensors, and time domain reflectometers. Tables 2, 3, and 4 of ref. [61] list the bridges with active or past fixed scour systems. Portable devices include sounding rods, sonars on floating boards, scour boats, and scour trucks. They are more cost-effective than fixed instruments because they can be transported from one bridge to another, and can therefore be used in multiple bridges. A third approach based on visual inspection is performed at standard regular intervals and can include increased monitoring during high flow events (flood watch), land monitoring, and/or underwater inspections. Both portable devices and visual inspections cannot be carried out during storms. When there is a high-flow event, the scour hole that is formed is often filled in during the receding stage as the stream flow returns to normal [61]. Hunt [61] summarized the response of a survey submitted to U.S. state DOTs. One of the concerns about the scour monitoring devices is the difficulty of maintenance and repairs to the scour monitoring systems and the damage caused to the systems by debris flows and accumulation, vandalism, and corrosion.

Various studies in bridge safety evaluation revealed that foundation scour is the major cause of bridge failure. Specifically, Lagasse et al. [62] noted that scour-related issues account for 60% of bridge failures in the United States, i.e., scour is the primary cause of bridge failure in the United States where more than 20,000 highway bridges are rated “scour critical” [61]. Thus, the understanding of damages and degradation caused by scour is important to make decisions for bridge maintenance and repair. Bridge failure caused by scour has been investigated by many researchers worldwide.

In 2005, a report by Walker and Hughes discussed the routine scour monitoring of three (3) bridges in Wisconsin, namely the County Highway B Bridge on the Crawfish river, the Wisconsin Highway 35 bridge in Tank Creek, and the Balsam Road Bridge over the Big Eau Pleine river. With regard the first bridge, Walker and Hughes [63] reported that a manual monitoring system was deployed and consisted of two manual wire-weight gages that were installed on the upstream rail of the bridge. Field data were collected monthly from March to May in 2003 and from May to June in 2004. The scientific literature regarding these three (3) rural bridges is scarce.

The Balsam Road Bridge was instrumented with two Datasonics PSA-916 sonar transducers on the upstream and downstream edges of one pier. A Campbell Scientific CR10 datalogger was connected to transducers to record data every 15 min. The instrumentation system ran from June 1998 to September 2001.

One pier of the Wisconsin Highway 35 Bridge spanning Tank Creek was instrumented with a Datasonics PSA-916 sonar connected to a Campbell Scientific CR10 datalogger. In addition, a Kellor KPSI Series 760 SDI-12 0–5 psi depth sensor was installed to measure the stream stage. The instrumentation system started operating in April 2000. Selected recording results were analyzed and the conclusion was that measurements ha some uncertainties that would affect accuracy, but which were within limits for scour monitoring.

Hunt [61] presented a report about the current state of practice for fixed scour bridge monitoring by performing a literature review, surveying the state transportation agencies, and conducting a few interviews. Thirty-seven U.S. state Departments of Transportation responded to the survey. Information about the other thirteen states was obtained from the literature review. Thirty-two U.S. states used fixed scour monitoring instrumentation at some point. Hunt [61] identified a total of 120 bridge sites that are using or have employed fixed scour monitors. The monitoring systems used by the states, with the exception of time domain reflectometry, are described in the current FHWA guidelines on scour countermeasures and monitoring, Hydraulic Engineering Circular 23.

In what follows, some studies posterior to Hunt [61] are summarized for the sake of completeness and to guide the interested readers in the subject of scour monitoring and assessment.

Foti and Sabia [64] investigated the influence on the dynamic response of a bridge in Northern Italy due to foundation scour. Two methods were proposed to evaluate the use of monitoring traffic-induced vibrations as an indirect method to infer foundation scour. For bridge span, a modal identification method based on the ARMAV technique [65] was proposed. For the piers, as modal identification is not effective, the dynamics response caused by traffic was used instead. Both methods were evaluated by using field data collected before and after the retrofit of the bridge pier.

Briaud et al. [66] evaluated various scour monitoring instrumentations including the use of accelerometers by conducting both laboratory and field experiments. In the laboratory trial, accelerometers and tiltmeters showed a good potential for bridge condition evaluation. The Fourier transform of the acceleration data and the ratio of root mean square values of acceleration in two different directions were shown to be good indicators to monitor the progress of scour. While the laboratory data showed good results, field experiments were corrupted by environmental factors including noise.

Hussein [67] investigated numerically the scour effects on the supports of a model-scale bridge and determined that, while the vertical mode shapes of the bridge are not sensitive to scour, the horizontal mode shapes have significant sensitivity to scour. To verify the numerical analysis result, the experiment was conducted on a real structure under three (3) scour scenarios. The Chicken Road Bridge (a two-span highly skewed bridge) in Lumberton, North Carolina was investigated numerically to verify the applicability of the proposed scour detection technique on a real bridge. The experimental results verified the numerical analysis; otherwise, the results showed that three (3) damage indicators were able to determine scour locations. Although the laboratory results showed significant promise for scour monitoring, more work is needed to make it practical for the actual bridge structures. Hussein [67] concluded that:

- The mode shapes relative to vertical displacements and the corresponding natural frequencies did not show significant changes due to scour. Therefore, they could not be used in scour detection, but they may be effective at detecting damages in the bridge superstructure as seen in Ch. 4.
- The 1st, 3rd, and 5th horizontally displaced mode shapes and their corresponding natural frequencies were successful in identifying the existence of scour. The natural frequencies of the significant mode shapes decrease as the natural frequencies increase due to the reduction in the flexural stiffness of the intermediate piles. Other mode-shapes, such as the 2nd and 4th, were insensitive to scour due to the presence of a stationary node at the location of scour.
- The curvature for the first five horizontally displaced mode shapes was successful in identifying the exact location of some of the scour cases considered.
- The magnitude of deflection increases as the scour level increases due to the decrease in the flexural stiffness of the piles. The absolute difference in the flexibility-based deflection from the unscoured case was calculated for various scour cases. The difference in the absolute difference in the deflections was able to identify the exact location of damage for the symmetrical scour cases and the damaged zone for the unsymmetrical and braced scour cases.

Lin et al. [68] created a finite element model with soil spring to simulate the relationship between the fundamental frequency and the embedded depth of the bridge column. This relationship was used to develop a scour detection algorithm based on ambient vibration of the superstructure. A set of laboratory experiments were conducted to verify the algorithm.

Based on the works presented in refs. [66,67], Prendergast et al. [69] proposed a method to detect and monitor scour development based on the changes in the foundation dynamic response caused by scour. A laboratory experiment was conducted with a 1.26-m pile installed in a sand box, where scour was modeled by removing the sand progressively. The pile was subjected to an impulse load at the top and the acceleration response was recorded by accelerometers. A Fourier transform was used to convert data into the frequency domain. A numerical model was calibrated by using the laboratory data to obtain a good matched model. The field experiment was conducted with the promising results from laboratory and numerical models. A full-scale pile which was driven into dense sand was tested with a similar method to the laboratory experiment. A good match between field test and numerical prediction was obtained.

Kong et al. [70] investigated static and dynamic responses of a single pile with scour effects. Three (3) possible methods for scour detection were proposed based on modal frequency change, bending moment profile, and modal strain profile. These methods were validated by a laboratory test.

Chen et al. [71] proposed a foundation scour evaluation method based on the ambient vibration measurement obtained from the superstructure of the cable-stayed bridge. Various girder and local pier modal frequencies were identified and an FE model based on the original design parameters was developed to perform modal analysis. Based on the measured and model results, the best support boundary conditions were identified as well as the optimal soil stiffness. Subsequently, the local pier scour depth can be estimated by changing the depth of the soil to fit the local frequencies.

### 2.6. Impact Effects

Bridge vibration caused by external impacts is another critical factor that needs to be considered for some structures. The impact of heavy vehicles with the superstructure or collision of ships and barges on piers and pylons cause spikes in vibration but may also cause permanent significant damage to structures. This is of critical interest for those bridges crossing heavy fluvial traffic. As such, some researchers have investigated the effects of impact events on bridge condition. As done for scour, this section presents a brief overview about the effects of impacts.

El-Tawil et al. [72] used an inelastic transient finite element simulation to investigate the collision between vehicles and bridge piers. Two different types of truck models were considered, namely a 14-kN Chevy truck, representing light trucks, and a 66-kN Ford truck, representing medium weight trucks. In addition, two different bridge/pier systems were simulated with approach speeds ranging from 55 to 135 km/h. The simulations showed that, in general, the peak transient forces are much higher than the AASHTO-LRFD collision design force at the time of the study. However, since the peak forces act for a short duration, equivalent static forces were computed to serve as a measure of “design” structural demands during collision. The computed equivalent static forces were also significantly higher than the AASHTO-LRFD design force for a number of simulations. These results imply that the AASHTO-LRFD design provisions (current in 2005) could be unconservative for feasible crash scenarios such as those considered in the study.

Song et al. [73] proposed an overnight collision detection and evaluation system for concrete bridge girders using piezoceramic transducers. A model concrete girder was used to conduct an impact test and health monitoring test with three piezoelectric transducers. An electric circuit was designed to detect the impact and activate a digital camera to take photos of colliding trucks. The PZT output, being proportional to the physical impact of the truck on the structure, can be used to predict the health of a structure. Impact levels can also detect the growth of cracks inside concrete structures when the structure is gradually damaged in repeated impact tests.

Yun et al. [74] investigated the effect of collision of a cargo ship with the Vincent Bridge in Los Angeles. They conducted a forensic study to evaluate the structural condition of the bridge before and after collision. A health monitoring system was installed and acceleration data were analyzed. Time-history records of the bridge oscillations before, during, and after the accident were analyzed using multi-sensor identification approaches based on the Natural Excitation Technique (NExT) in conjunction with the Eigensystem Realization Algorithm (ERA). These processing approaches served to extract the modal characteristics of the bridge. Yun et al. [74] determined that the analysis carried out in the study can provide the owners with forensic tools that enable reliable and rapid assessment of extreme events.

## 3. Wireless Sensor Technologies and Sensor Drift

The cost of traditional wired SHM systems, due in part to cabling networks, is detrimental for the deployment of high-density sensor systems or for usage in long-span bridges. SHM using wireless sensors can overcome the limitations of traditional wired methods with many attractive features such as wireless communication, on-board computation, battery power, ease of installation, and so on. Many groups worldwide, including researchers at the University of Illinois at Urbana Champaign [75,76,77,78] have successful implemented wireless sensor technologies for SHM and demonstrated the efficacy of such technologies in measuring structural acceleration, strain, and displacement responses over full-scale applications [79].

Wireless smart sensing (WSS) are devices that have sensor, microprocessor, radio frequency transceiver, memory, and power source integrated into one small-sized unit and are characterized by their capabilities of sensing, computation, data transmission, and storage, all achieved by a single device. They are increasingly considered as SHM platforms because they represent an alternative to their wired counterparts. WSS are attractive because their cost is lower cost (including cost for labor) due to the absence of long cables and due to the widespread production of micro-electro-mechanical sensors. The wireless communication allows flexible network topology and enables a decentralized monitoring scheme as opposed to the centralized scheme of wired systems [80].

Smart sensing platforms generally feature several characteristics: (1) on-board CPU; (2) small size; (3) wireless communication and data transmission; and (4) low-cost. Many WSS platforms have been developed and applied in SHM. They include the Mica series, iMote series, and Xnode. The Xnode is an advanced wireless sensing platform with several critical features such as reliable wireless communication, high-fidelity analog-to-digital converter, expandable data storage, high-precision synchronized sensing, user-configurable middleware software library, automated long-term operation of wireless network, and so [81]. Based on advanced wireless sensing platforms, many kinds of accompanying sensor boards have been developed to interact with the wireless sensing platforms for achieving diverse sensing capabilities. Because the measurement environment, frequency range, and budget may be different for the purpose, various types of external sensor boards for WSS were developed for acceleration, high-sensitivity strain, and environmental measurements [82].

Noel et al. [83] presented a comprehensive review of WSNs for SHM applications, outlining the: (1) algorithms used in damage detection and localization, (2) network design challenges, and (3) future research directions. Solutions to network design problems such as scalability, time synchronization, sensor placement, and data processing were compared and discussed. The work by Noel et al. (2017) is one of the most recent papers on the subject and follows the other excellent reviews from Lynch and Loh [84], and Aygün and Gungor [85].

More recently, Abdulkarem et al. [86] reviewed wired and wireless sensor SHM systems including wireless sensor node architecture, communication technologies, and operating systems. The review included the state-of-the-art academic and commercial wireless platform technologies used in laboratory and field tests. The key challenges associated with WSN for SHM were identified.

The reliability of the sensors is crucial for a successful SHM implementation and a trustworthy complement to annual inspections. As sensors age along with the structure they aim to monitor, sensors may experience a drift phenomenon. Drift is a slow, often linear or exponential decrease in accuracy that can at times go unnoticed if not appropriately monitored. If a sensor becomes too inaccurate, it can trigger false positives or (in the worst case scenario) false negatives. False positives would require on-site inspection to verify the alarms. False negatives would leave critical damage unnoticed until the next cycle of bridge inspection.

Engineers and SHM specialists are therefore challenged with the development of methods and strategies to alleviate or eliminate drift due to sensor age without becoming a costly practice that nulls the economic advantages of wireless sensors with respect to wired technology. As a matter of fact, despite that wireless sensors have become relatively inexpensive, the cost in labor to replace them can be very high, and in some cases is an incredibly high safety risk.

Currently, there is not a set standard for ways to correct this drift; however, various methods have been researched. One of these methods is to cluster sensors in regions, rather than have a single sensor, and to evaluate the detections of all sensors in the cluster. By determining a uniform baseline from this data, sensors are calibrated back to a determined “zero-line” and establish the detectable threshold from there. This can be done either manually using remote computers or by utilizing auto-calibrating sensors. The approach also relies on the ability to recognize data that are skewed and reject them from the results in order to prevent errors [87].

Despite nearly two decades of developments, wireless-based SHM systems still face other challenges besides drift. These challenges include but are not limited to powering the system and therefore harvest energy, limited communication bandwidth and range, data loss, time synchronization, and signal length [80,83]. Note that in the context of this review paper, synchronization is separate from drift.

Issues related to synchronization were reviewed and addressed by Li et al. [80]. Synchronization errors may occur because each smart sensor in the network has an independent processor with its own local clock which is not necessarily synchronized with the clocks of other sensors. Moreover, even with clocks that are perfectly synchronized, the data may lack synchronization because: (1) the sensors start sensing at different times due to random variations in the processing time in the sensor board driver; (2) the low quality of crystals may cause differences in the sampling frequencies among the nodes; and (3) the sampling frequency for each individual sensor node can fluctuate over time because of jitter [80]. When data are not synchronized, there is a shift in the phase information, which some algorithms consider as damage indicators. When modal analysis is used and high frequency vibrations are used to detect damage because such vibrations are more sensitive to local defects, the accuracy of time synchronization at high frequency is pivotal. For example, a 1 *millisec* synchronization error between two measured accelerations will result in a 3.6° error in phase angle at 10 Hz and a 36° error at 100 Hz. So, if the damage detection algorithm linked to the SHM system is based on mode shapes, the mode shape error due to phase angle errors can lead to false positives.

The scientific literature contains several time synchronization protocols for WSSNs and the interested readers are referred to [80] for a brief overview and for a few solutions experimented on a Korean bridge and on the Arsenal Bridge in the U.S. The monitoring of the latter added extra challenges related to the extended sensing duration required to fully capture a transient event. For example, the monitoring of an entire vibration response during a swing event requires 10 min of data at 50 Hz (30,000 data points).

Clock drift is also caused by temperature variation to which a sensor may be exposed during the day due to exposure to direct sunlight or other weather-related factors, or because of heat generated by the processor itself. It has been shown that temperature change can cause nonlinear clock drift which poses an additional challenge for synchronized sensing in SHM.

Sensing for SHM is characterized by much more sampled data points than, for example, temperature measurements. For example, under a given frequency bandwidth, more data points provide higher resolution once the data is converted into the frequency domain, and therefore, higher accuracy of estimated modal frequencies can be achieved. The requirement for longer sensing duration needed to extract meaningful information of structural characteristics poses challenges related to power, board memory, and data transmission. Longer recordings are instead required to capture the complete record of the forced vibration caused by a train crossing the bridge, which takes about 10 min. Extended sensing duration may exacerbate the problem of clock drift (skew), which is a phenomenon where two clocks drift away from each other because of differential clock speed. Even though the clocks were accurately synchronized when sensing started, they can drift away from each other during sensing and cause errors in timestamps, which in turn leads to synchronization error in the sampled data. Li et al. addressed this problem by implementing clock skew compensation [80].

## 4. Conclusions

This article discusses data analysis methodologies and issues associated with wired and wireless sensors in the framework of bridge health monitoring. The scope of the article is to provide a holistic view on the challenges related to the extraction of meaningful data from sensors bonded, bolted, or embedded on bridges. Owing to the breadth of the topic and the number of bridges currently instrumented worldwide, the attention of this study was mainly on U.S. bridges and instrumentation programs developed over the last 20 years. For this reason, many different aspects are covered, but some topics are described in detail like “scour” or “time and temperature-dependent factors” while others (“corrosion evaluation”) are mentioned in shorter paragraphs like “corrosion evaluation”. The article complements a recent study by the authors [8]. With respect to ref. [8], which focused on methodologies and objectives of the bridge instrumentation programs in the U.S., this paper focuses primarily on methods to evaluate structural parameters and detect structural irregularities using data from varying instrument types, and data validation techniques to assess the accuracy of the recorded data including data collected using wireless sensors. Overall, the following conclusions are drawn:

1. Owing to the size of the structures being involved and owing to the nature of the degradation processes, the analysis and the comprehension of the field data is more successful when complemented with robust finite element modeling.
2. Whenever finite element modeling has supplemented the SHM protocols, the latter ones are deemed the ones providing the accurate results and therefore the models are calibrated to “match” field data.
3. Time-dependent phenomena such as creep and shrinkage of concrete may affect structures and therefore may affect sensor data.
4. Temperature plays a detrimental role in the static (strain) and dynamic (modal) analysis of bridges. Any robust SHM strategy cannot disregard the effects of temperature and proper compensation methods are necessary to extract live load or long-term effect from daily measurements.
5. Factors such as snow or water were not found as potential factors influencing field data.
6. Despite that wireless sensing is increasingly used in bridge health monitoring, there are still technical challenges that prevent its exclusive use in lieu of conventional wired systems.
7. As bridges have very little in common with each other and almost any new bridge is unique, it is difficult to design a uniform SHM paradigm valid for any bridge. What is adequate for some may not be adequate for another. This complication increases when structures are modeled but damage can only be simulated numerically but not (logically) induced experimentally.

Owing to the scope of the manuscript, the literature reviewed in this study should not be considered comprehensive of all the work conducted worldwide. A query like “structural health monitoring” AND “bridges” AND “United States” was the starting point of the study. While peer-review articles and technical reports submitted to U.S. Federal Agencies and Departments of Transportation were included, patents, conference proceedings abstracts, and advertisement material were excluded. In addition, other criteria of exclusion were all those methodologies related to remote sensing such as unmanned aerial vehicles, video-based, infrared cameras, GPS, and advanced cloud computing (see for example ref. [88]) were not considered to keep the review within reasonable size. Likewise, issues related the limitations of the short lifetime of wireless sensor networks due to high power consumption and solutions such as energy harvesting were not discussed in detail. Possible solutions could be using power by harvesting energy from vibration or renewable energy (solar) or by using multi-agent-based routing able to traverse the sensor nodes mounted onto a bridge using multi-hop communication. Examples of such solutions can be found in [89], although they are not applied on existing bridges.

For those readers interested in gaining more insights about algorithms and step-by-step procedures on how the SHM systems were designed based on bridge location and importance, size of the object, type of material used for the super-structure, etc., they are referred to the complementary review [8] recently published by the authors.

## Figures and Tables

**Table 1:** Bridges in the United States instrumented with sensing systems prior to 2011 according to Table 1.1 of ref. [7].

#	Name	Location	Type
1	Golden Gate	San Francisco, CA	Suspension
2	Fred Hartman	Houston Ship Channel, TX	Cable-stayed
3	Sunshine Skyway	Tampa Bay, FL	Cable-stayed
4	Quincy Bayview	West Quincy (MO)—Quincy (IL)	Cable-stayed
5	Commodore Barry	Chester (PA)—Logan Twn (NJ)	Truss
6	Ironton-Russell ^1^	Ironton (OH)—Russell (KY)	Truss
7	New Benicia Martinez	San Francisco, CA	Box
8	Saint Anthony Falls I-35W	Minnesota, MN	Box
9	North Halawa Valley	Oahu, HI	Box

^1^ This bridge closed in 2016 and was replaced by a new cable-stayed bridge. This new bridge was opened on 23 November 2016 but it is unclear if it is under surveillance with an active SHM system.

## Data Availability

No new data were created in this study. Data sharing is not applicable to this article.
